# Construction and validation of an eight-gene signature with great prognostic value in bladder cancer

**DOI:** 10.7150/jca.38741

**Published:** 2020-01-17

**Authors:** Xin Yan, Xun Fu, Zi-Xin Guo, Xiao-Ping Liu, Tong-Zu Liu, Sheng Li

**Affiliations:** 1 Department of Urology, Zhongnan Hospital of Wuhan University, Wuhan 430071, China.; 2 Department of Biological Repositories, Zhongnan Hospital of Wuhan University, Wuhan 430071, China.; 3 Human Genetics Resource Preservation Center of Hubei Province, Wuhan 430071, China.

**Keywords:** bladder cancer, LASSO, WGCNA, grade, survival, gene signature, nomogram, overall survival, prognosis

## Abstract

Bladder cancer (BC) is one of the most common malignancies in urinary system with a common malignancy in urinary system with a high mortality and recurrence rate, so we attempt to construct a gene signature to predict the prognosis of BCs. We initially established a co-expression network by performing WGCNA analysis and further identified magenta module as key module (P = 8e-05, R2 = 0.4). Subsequently, we screened 12 genes associated with survival from the key module, which were selected to construct an eight-gene signature by establishing a LASSO Cox model. Moreover, we reckoned the risk score (RS) of each sample, through which we could divide samples into two groups (the high-risk and low-risk groups) and verify the signature, in the training set and 3 validation sets (internal test set, GSE13507and E-MTAB-4321). This signature could distinguish between the high- and low- risk patients well (survival analysis: P = 0.015; AUC: 0.61 at 1 year, 0.61 at 3 years and 0.61 at 5 years). In the validation sets, this signature also showed good performance, which was consistent with the training test. Furthermore, we plotted a nomogram to predict the possibility of the overall survival (OS) and three calibration curves to predict the effectiveness of the nomogram, which suggested good value and clinical utility of the nomogram. In conclusion, we established an eight-gene signature, which was probably effective in the prediction of prognosis of patients with BC.

## Introduction

BC is the tenth most common type of cancer worldwide, with approximately morbidity of 3.0% and mortality of 2.1% 1. In addition, BC is a malignancy with a poor prognosis and a high recurrence rate (30-70%), the five-year survival rate of which is approximately 50-70% as reported 2. Thus, many patients may lose the best opportunities to be diagnosed and cured because of the high morbidity and mortality as well as the poor prognosis, which means that some new methods to diagnose and predict the prognosis of BC are required.

We have noticed that some studies identified novel prognostic biomarkers for BC by using bioinformatics methods 3, 4, which means it is promising to utilize biomarkers to predict the prognosis of patients 5. But almost all these studies only explore the predictive value of a single biomarker 6. Actually, the effect of just a single biomarker for predicting prognosis is probably not sufficient in BC. Thus, by performing least absolute shrinkage and selection operator (LASSO) Cox regression model, the aim of this study was to develop and validate a more effective and useful multi-gene signature and further establish a prognostic nomogram based on several biomarkers obtained through the up-front bioinformatics analysis, which might be of great and predictive value for the prognosis of BC patients.

## Materials and methods

### Data collection and data preprocessing

**Figure 1:**
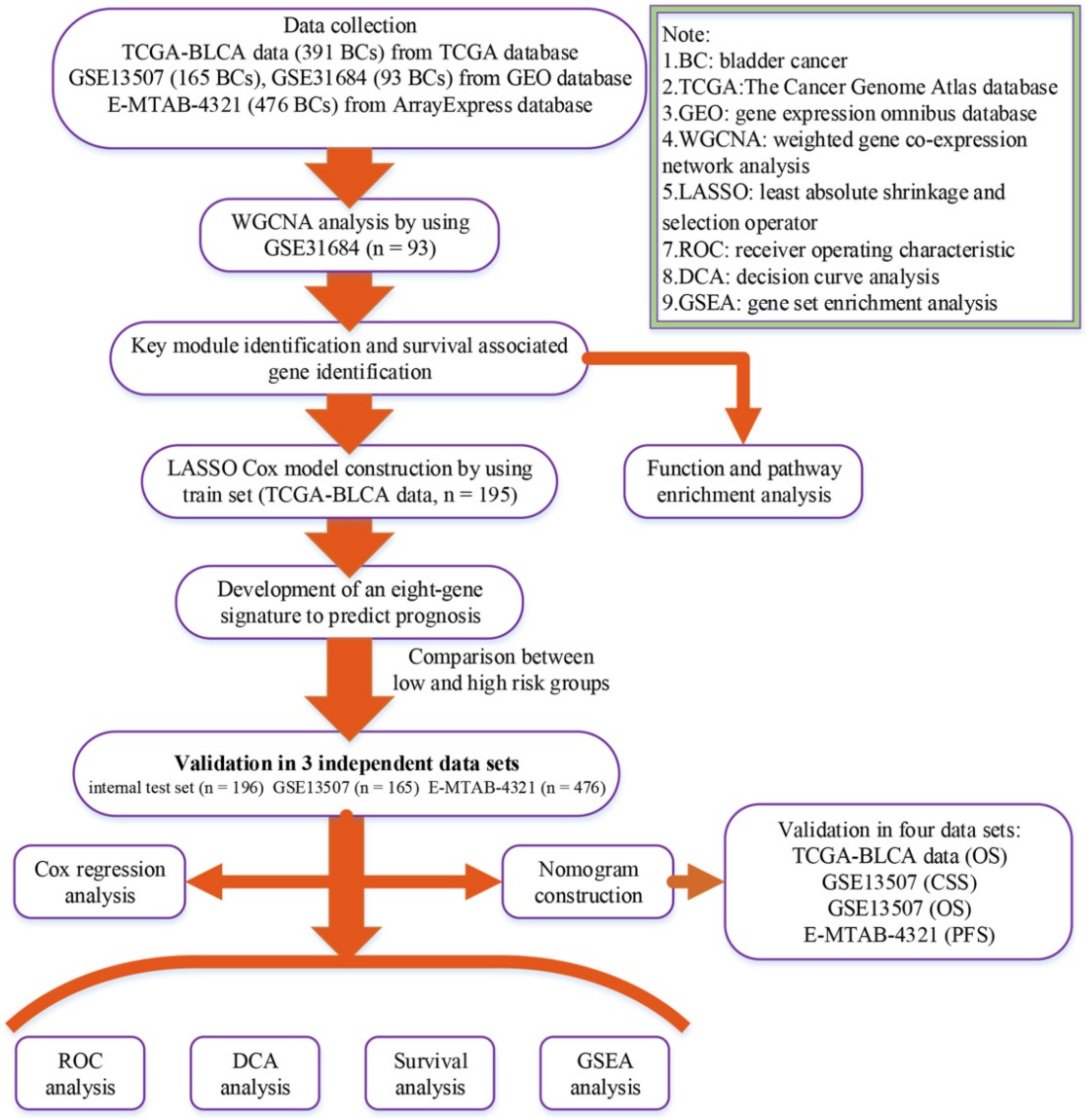
Flow chart indicating the process used to select target genes included in the analysis.

A flow chart of the study was shown in Figure 1. Data GSE31684 7, 8 including 93 BCs was downloaded from Gene Expression Omnibus (GEO) database (https://www.ncbi.nlm.nih.gov/geo/) for weighted gene co-expression network analysis (WGCNA), which was performed on Affymetrix Human Genome U133 Plus 2.0 Array (GPL570). We firstly annotated GSE31684 by transforming probes into genes and then calculated variances of genes across all samples. Only the top 5,000 most variant genes were picked out for constructing a co-expression network. Moreover, based on The Cancer Genome Atlas (TCGA) database (https:// gdc‐portal.nci.nih.gov/), the mRNA-seq expression profile and clinical information of BC patients were downloaded for LASSO Cox regression analysis. Totally 391 samples were included after getting rid of samples without complete survival information. 391 BCs were randomly assigned into two groups at the rate of 1:1. 195 samples formed the training set, which was used to construct a LASSO Cox model. The test set including 196 BCs was used to verify this model, which was regarded as an internal validation set. Furthermore, we also downloaded two other independent datasets GSE13507 9, 10 including 165 BCs from GEO database and E-MTAB-4321 including 476 BCs from ArrayExpress database (https://www.ebi.ac.uk/arrayexpress/) for external validation. “DEseq.2” 11 in R software was used for normalization and log2 transformation for TCGA-BLCA data displayed as count number. As for GSE13507 and GSE31684, we downloaded the Raw data and annotated the probes based on the corresponding annotation files. Then normalization and log2 transformation were performed by using R package “affy” 12. Normalized expression matrix of E-MTAB-4321 was directly downloaded from ArrayExpress database for subsequent analysis. Some important clinical features including age, gender, grade, and stage were available in Table 1.

### WGCNA analysis to screen out a key module

We initially checked the expression data profile of top 2,500 genes whether they fit the co-expression network construction by using sample network method. A sample was considered to be array outlier when its Z.Ku < -2.5, which was weeded out from the profile. Then based on R package “WGCNA” 13, we constructed a co-expression network. In order to classify genes into gene modules, three independent methods including manual (interactive) branch cutting approach, automatic single block analysis and 2 block analysis were used. In the process, we set a relatively large minimum module size (minClusterSize = 30) and a medium sensitivity (deepSplit = 2) for branch splitting. Furthermore, we identified key modules correlated with grade which interested us most through two methods. We firstly quantized the correlation between module eigengenes and traits, and further quantify the relationship by calculating Gene Significance (GS). Through the data processing, we got the average value of GS of all the genes in a module, called Module Significance (MS). The most positively relevant module was considered to be key module according to the results.

### Functional enrichment and pathway enrichment analysis

Gene Ontology (GO) enrichment analysis and Kyoto Encyclopedia of Genes and Genomes (KEGG) pathway analysis were performed for genes within the key module based on “clusterProfiler” 14 in R software. Gene sets with P value less than 0.05 were referred to be significantly enriched.

### Genes associated with survival identification

Having completed aforesaid identification of gene module, we preliminarily picked out genes according to the standard: |cor.geneModuleMembership| > 0.5 and |cor.geneTraitSignificance| > 0.2. Furthermore, we utilized overall survival (OS) analysis to pick out genes related to survival from those preliminarily picked genes by an online tool called Kaplan-Meier Plotter (https://kmplot.com/analysis/). Genes with significant *P* value (*P* < 0.05) in OS analysis would be thought as survival associated genes.

### Constructing and verifying a multi-gene signature

For the construction of LASSO Cox model, we extracted the expression data of survival associated genes based on the training set. We afterwards performed LASSO Cox regression analysis by R package “glmnet” 15, after which genes signature containing most helpful biomarkers for prognosis was obtained and then the risk score of each sample in all the datasets was calculated through the signature. Furthermore, we divided samples in all the datasets into high- and low- risk groups relying on their own median risk score. Then we performed survival analysis by using R package “survival” 16 and time-dependent (1-year, 3-year, and 5-year) receiver operating characteristic (ROC) analysis by using R package “timeROC” 17 in all the datasets (training set, internal test set, GSE13507, and E-MTAB-4321) to verify the prognostic value of the multi-gene-based classifier.

### Univariate and multivariate Cox regression analysis

To complete the Cox proportion hazard regression analysis, the multi-gene signature (using this signature as feature and the risk score as feature value) and other significant clinical features including gender, age, pathologic stage, and histologic grade were included in cox univariate analysis of overall survival (OS) by using TCGA-BLCA data, and elements in aforesaid analysis whose *P* value was less than 0.1 were brought into cox multivariate analysis. R package “forestplot” 18 was used to visualize the results.

### Nomogram construction and validation

Before the nomogram construction, cross-validation was performed to deal with over-fitting of the model. Package “rms” in R software was utilized to plot not only the nomogram but also calibrate curve which could test the nomogram. 45° line in the calibrate curve represented the best prediction. In addition, decision curve analysis (DCA) was conducted through R package “rmda” 19 to ensure if the multi-gene signature was clinically helpful. Moreover, we calculated the C-index (Concordance index) and AUC (area under curve, based on R package “pROC” 20) between the actual observation frequency and the predicted probability to estimate the accuracy of the nomogram. TCGA-BLCA was used for internal validation and GSE13507 and E-MTAB-4321 were used for external validation.

### Gene set enrichment analysis (GSEA)

We firstly reckoned the median risk score on the basis of aforesaid dataset GSE31684 (the dataset used for WGCNA analysis) for the reason of the understanding of underlying functions of the multiple-gene signature. Afterwards, 93 BC samples were split into two groups (high-risk group and low-risk group) through the median risk score. We set “c2.cp.kegg.v6.2.symbols.gmt”as the reference gene set, and further performed GSEA 21 among the two groups. KEGG signaling pathways were filtered by a cut-off criteria of nominal *P* < 0.05, |ES| > 0.6, and FDR < 25%.

## Results

### Key module identification

**Figure 2:**
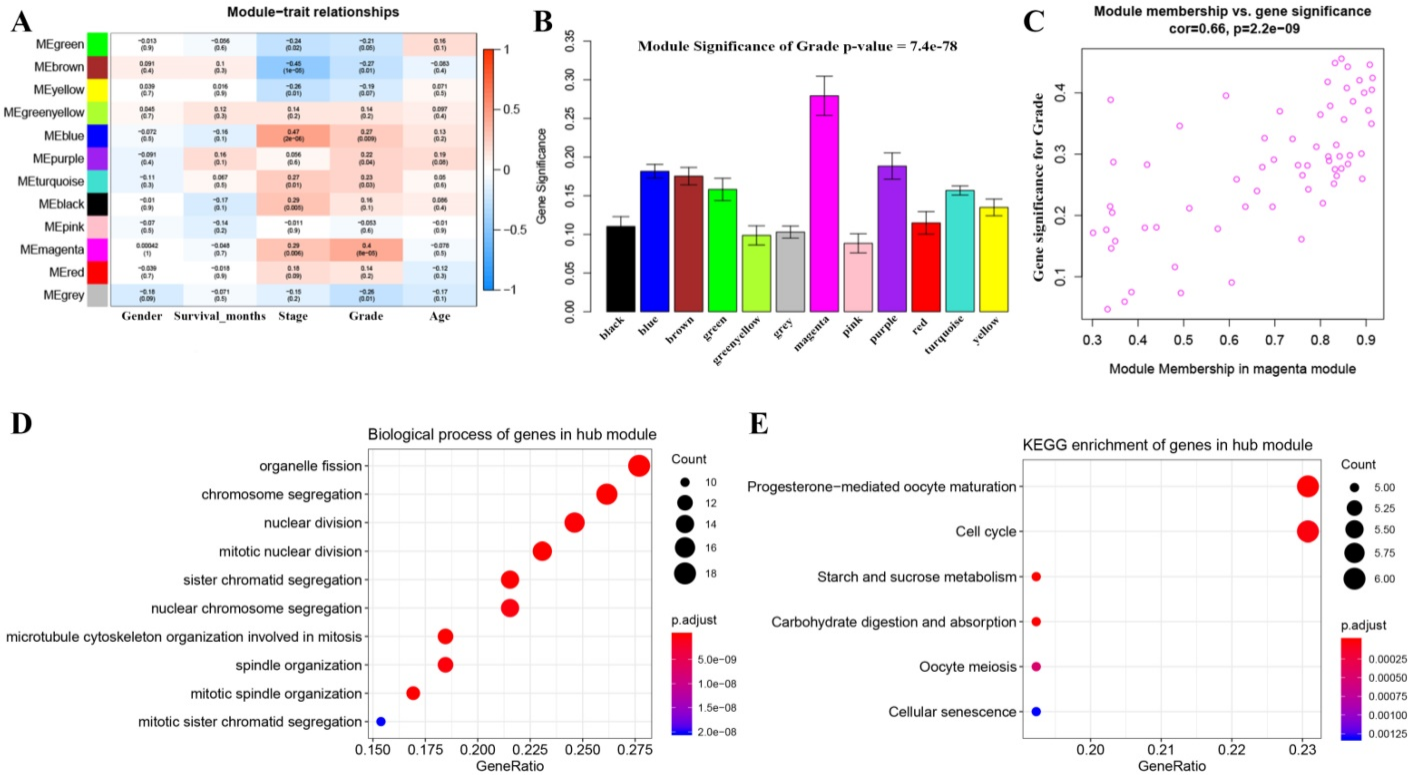
Identification of modules associated with the clinical traits of BC and bioinformatics analysis of genes in the hub module. (**A**) Heatmap of the correlation between module eigengenes and clinical traits of BC. (**B**) Distribution of average gene significance and errors in the modules associated with grade of BC. (**C**) Scatter plot of module eigengenes related to grade in the magenta module. (**D**) Biological process of genes in the hub module. (**E**) KEGG pathway enrichment of genes in the hub module.

3 outlier samples were weeded out by using sample network methods, totally 90 samples were used for constructing a co-expression network (Figure S1). As shown in Figure S2, beta (β) = 4 (scale free R^2^ = 0.85) was further set as the soft-thresholding for adjacencies calculation. Furthermore, 11 modules were identified in total by classifying genes into gene modules and merging modules (Figure S3). Genes without being classified into any other module formed the grey module, which was abandoned for further analysis. Among these modules, the magenta module was the most correlated one with grade positively (*P* =8e-05, R^2^ = 0.4) (Figure 2A), which was considered to be key module in this study. In addition, the MS of magenta module was higher than those of other modules suggested by Figure 2B-C. A network heatmap and a classical MDS plot were showed in Figure S4.

### GO and KEGG pathway analysis

Totally 137 biological processes (BPs) were shown in Table S1, which were enriched by genes in the key module. The top 10 BPs were organelle fission, chromosome segregation, nuclear division, mitotic nuclear division, sister chromatid segregation, nuclear chromosome segregation, microtubule cytoskeleton organization involved in mitosis, spindle organization, mitotic spindle organization, and mitotic sister chromatid segregation as shown in Figure 2D. Moreover, six KEGG pathways including progesterone-mediated oocyte maturation, cell cycle, starch and sucrose metabolism, carbohydrate digestion and absorption, oocyte meiosis, and cellular senescence were significantly enriched by genes in the key module suggested by Figure 2E.

### Survival associated gene identification

At first, we preliminarily selected 45 genes from the key module, which contained 65 genes originally, through the criterion:|cor.geneModuleMembership| > 0.5, and |cor.geneTraitSignificance| > 0.2. Moreover, we used Kaplan-Meier Plotter to preform OS analysis for the 45 genes, and 12 genes associated with survival were eventually identified for subsequent analysis. The results of OS analysis of the 45 genes were showed in Table S2.

### Developing an eight-gene signature for predicting OS

**Figure 3:**
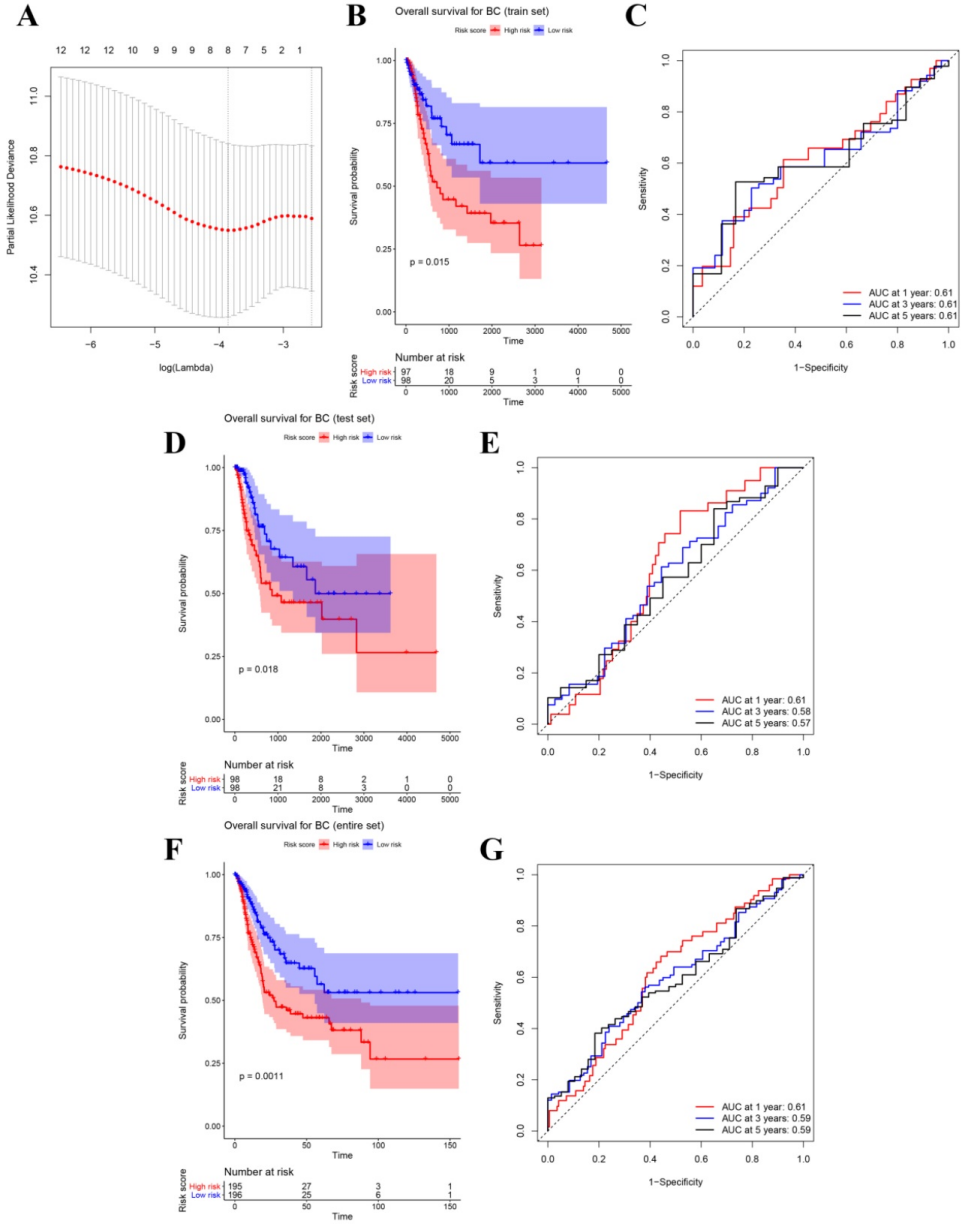
(**A**) Plot of partial likelihood deviance for the 12 genes associated with survival in the training set. Survival analysis of the association between risk score and overall survival time of BC in (**B**) training set, (**D**) internal test set, (**F**) entire set. Time dependent ROC analyses at 1,3, and 5 years in (**C**) training set, (**E**) internal test set, (**G**) entire set.

After identifying 12 biomarkers significantly correlated to survival of BCs, we further calculated the relative regression coefficient of the 12 genes by performing LASSO analysis. Eight genes including ASPM, C4orf46, CCNB1, DIAPH3, MLF1, MTFR2, NSUN6, and OIP5 were screened out to establish a multi-gene signature on account of the coefficient suggested by the LASSO model (Figure 3A). In addition, we also calculated the risk score (RS) of each sample by combining the relative expression levels (represented by relative expression values) and relative regression coefficients of the genes in the classifier as follows: RS = -0.135 × ASPM expression value - 0.142 × C4orf46 expression value + 0.632 × CCNB1 expression value + 0.105 × DIAPH3 expression value + 0.803 × MLF1 expression value - 0.456 × MTFR2 expression value - 0.287 × NSUN6 expression value + 0.05 × OIP5 expression value. Moreover, among the eight biomarkers, CCNB1, DIAPH3, MLF1, and OIP5 had positive coefficients meanwhile ASPM, C4orf46, MTFR2, and NSUN6 had had negative coefficients. According to the median-RS (RS = 7.373) in training set, 195 samples were divided into high-risk group (n = 97) and low-risk group (n = 98). The result of survival analysis indicated that high-risk group had a poor OS of BC patients (*P* = 0.015, Figure 3B). In the training set, the AUC values of the eight-gene signature were 0.61 at 1 year, 0.61 at 3 years, as well as 0.61 at 5 years suggested by Figure 3C.

### Prognostic value of the eight-gene signature

**Figure 4:**
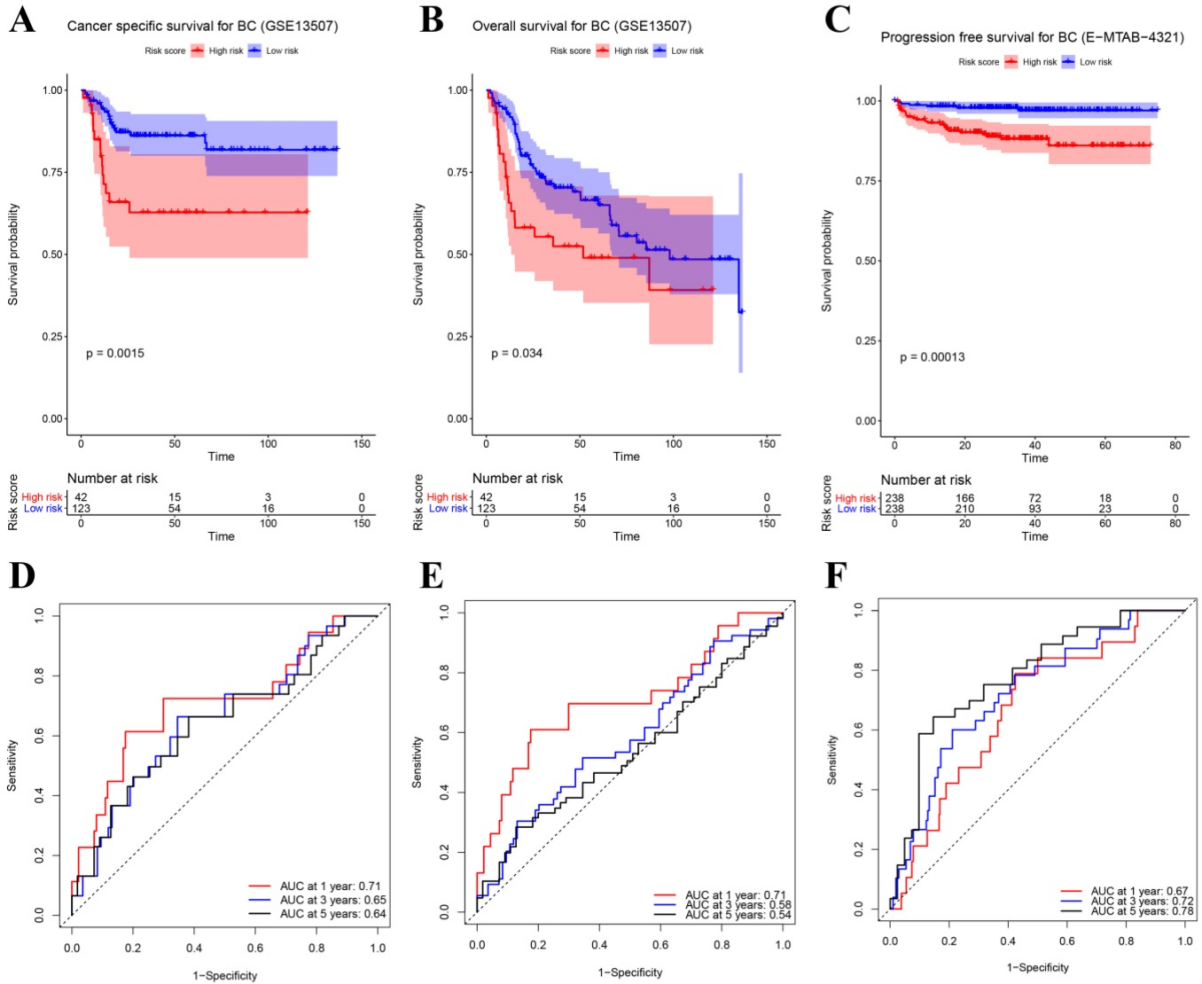
(**A**) Survival analysis of the association between risk score and cancer-specific survival (CSS) time of BC in GSE13507. (**B**) Survival analysis of the association between risk score and overall survival (OS) time of BC in GSE13507. (**C**) Survival analysis of the association between risk score and progression-free survival time of BC in E-MTAB-4321. Time dependent ROC analyses at 1,3, and 5 years in (**D**) GSE13507-CSS, (**E**) GSE13507-OS, (**F**) E-MTAB-4321.

**Figure 5:**
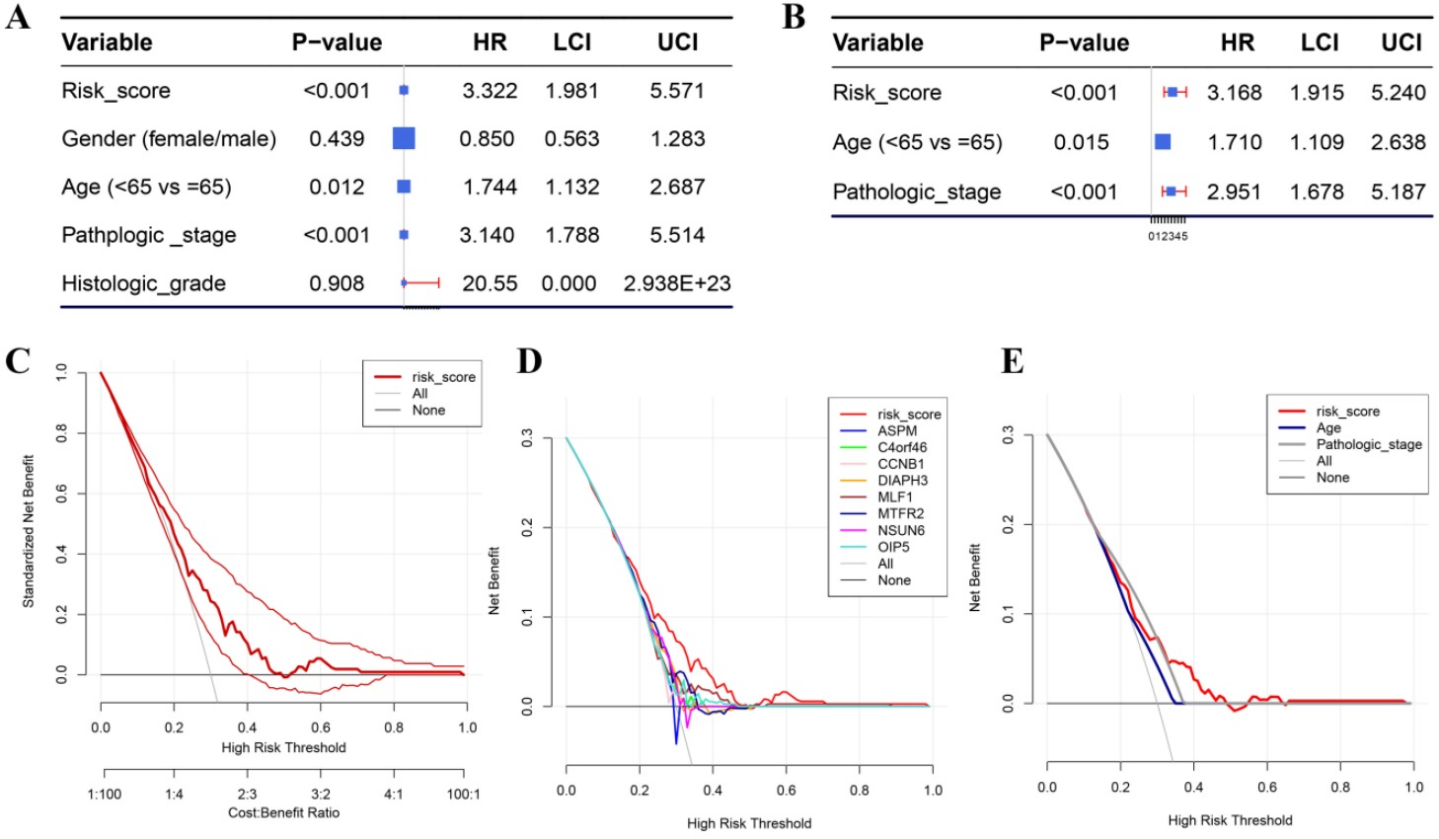
(**A**) Forest plot summary of analyses of OS univariate analysis of Risk score, gender, age, pathologic stage and histologic grade by using TCGA-BC data. (**B**) Forest plot summary of analyses of OS univariate analysis of Risk score, age and pathologic stage by using TCGA-BC data. DCA for assessment of the clinical utility of the 8-gene signature (**C**), single biomarkers (**D**), and clinical factors (**E**). The x‐axis represents the percentage of threshold probability, and the y‐axis represents the net benefit. DCA: decision curve analysis.

Besides the training set, we also produced 3 validation sets (internal test set, GSE13507 and E-MTAB-4321) to confirm the results we obtained from the training set. With the same methods we mentioned above, we calculated the risk score of each sample in these validation sets and the median-RS in each validation set was set as the cut-off criteria to split samples into high- and low- risk groups (median-RS: internal test set: 7.370; GSE13507: 5.558; E-MTAB-4321: 8.193). According to the result of survival analysis, high-risk group was more correlated to a lower survival rate in internal test set (*P* = 0.018, Figure 3D), entire set (consisting of the internal test set and training set, *P* = 0.001, Figure 3F), GSE13507(Cancer-specific survival (CSS): *P* = 0.002; OS: *P* = 0.034, Figure 4A-B) and E-MTAB-4321 (Progression-free survival (PFS): *P* < 0.001, Figure 4C). Moreover, the prognostic accuracy of the eight-gene signature in the internal test set was 0.61 at 1 year, 0.58 at 3 years, and 0.57 at 5 years as shown in Figure 3E (entire set: 0.61 at 1 year, 0.59 at 3 years, and 0.59 at 5 years (Figure 3G)). The AUC values of the multi-gene signature for CSS in GSE13507 were 0.71 at 1 year, 0.65 at 3 years, and 0.64 at 5 years meanwhile 0.71 at 1 year, 0.58 at 3 years, and 0.54 at 5 years for OS (Figure 4D-E). In addition, as shown in Figure 4F, the prognostic accuracy of the 8-gene signature in E-MTAB-4321 was 0.67 at 1 year, 0.72 at 3 years and 0.78 at 5 years. Furthermore, the eight-gene signature and significant clinical factors were included in the univariate Cox analysis. Figure 5A indicated that risk score (Hazard Ratio(HR) = 3.322, 95%CI: 1.981-5.571, *P* < 0.001), age (HR = 1.744, 95%CI: 1.132-2.687, *P* = 0.012) and pathologic stage (HR = 3.140, 95%CI: 1.788-5.514, *P* < 0.001) were influential features of OS among those important factors, which were used for multivariate Cox analysis. The result of multivariate Cox analysis suggested that these three variables were still associated with OS of BC patients even through the adjustment of other features (Figure 5B). In addition, the DCA analysis indicated that this eight-gene signature showed good potential for clinical application whatever the Threshold Probability (Pt) as shown in Figure 5C. Furthermore, although this signature also showed better performance than any other single biomarker (Figure 5D), we could not distinguish this signature from age and pathologic stage well when Pt was approximately less than 0.35 (Figure 5E).

### Nomogram construction and its clinical utility

**Figure 6:**
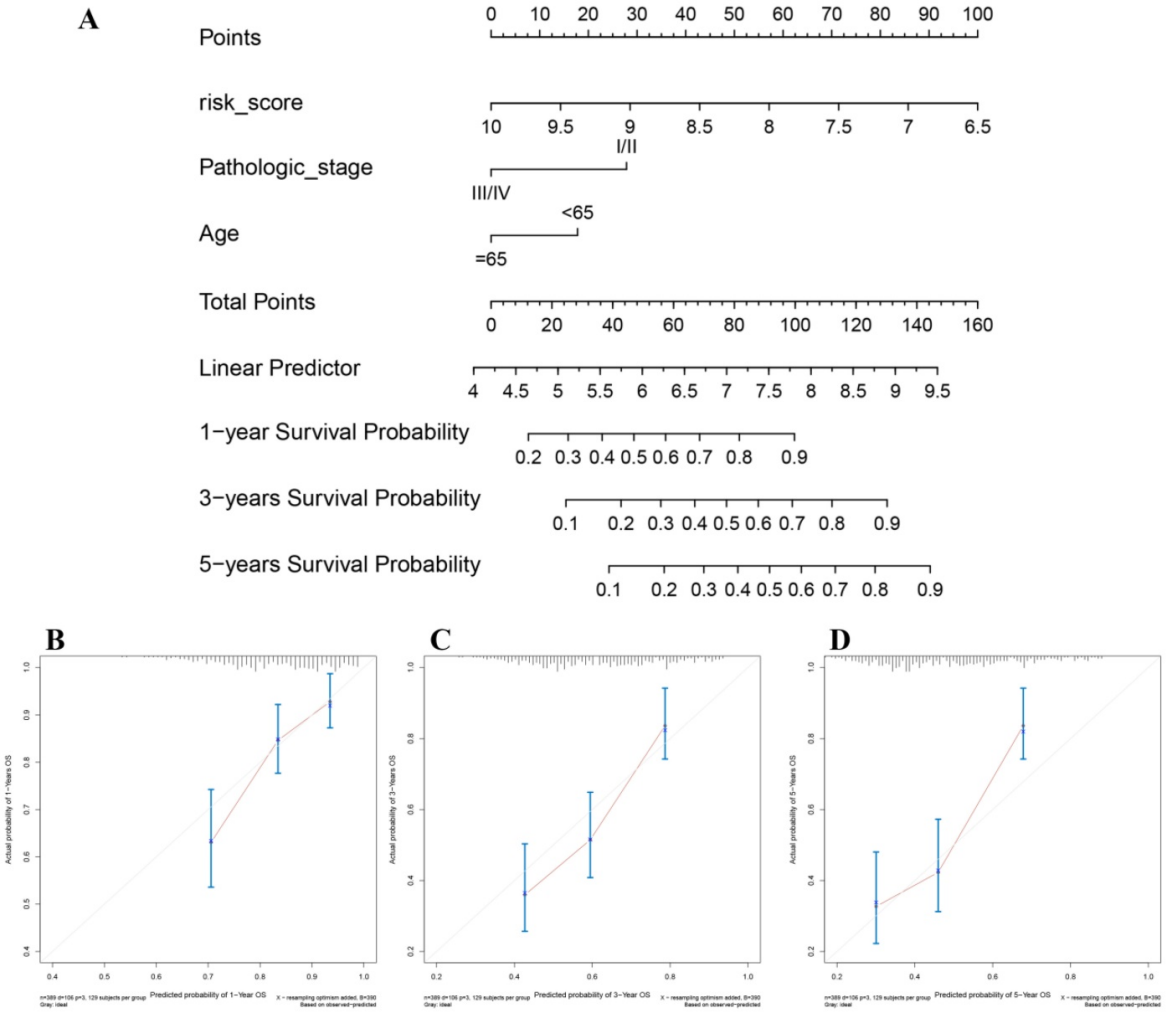
The nomogram for predicting proportion of patients with 1-, 3- or 5-year OS (**A**). The calibration plots for predicting 1- (**B**), 3- (**C**) or 5- (**D**) year OS.

**Figure 7:**
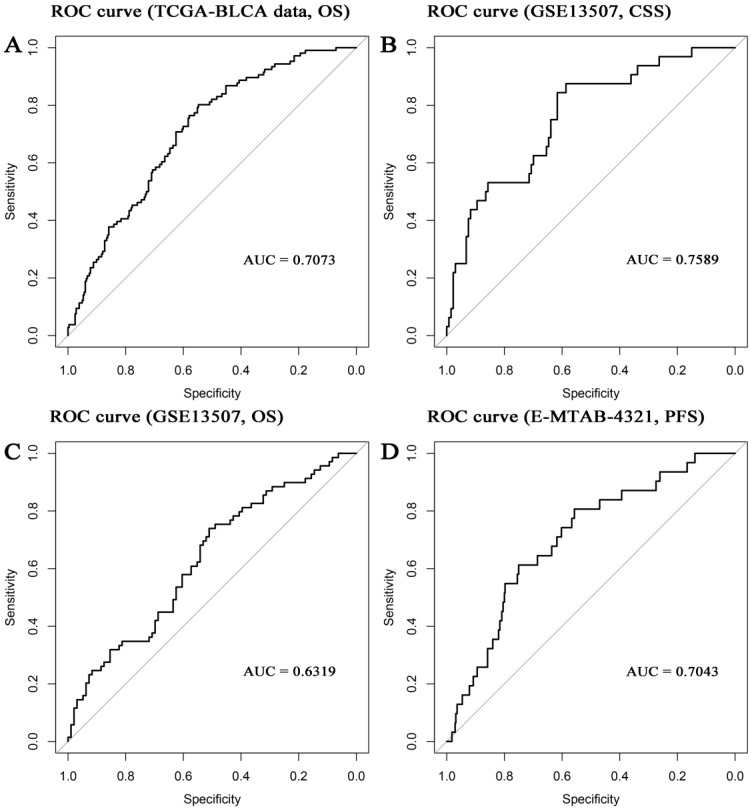
Receiver operating characteristic (ROC) curves and area under the curve (AUC) statistics to evaluate the diagnostic efficiency of the nomogram in TCGA-BLCA data (**A**, overall survival (OS)), GSE13507 (**B**, cancer-specific survival (CSS)), GSE13507 (**C**, OS), and E-MTAB-4321 (**D**, progression-free survival (PFS)).

In the present study, the 8-gene signature, pathologic stage, and age were incorporated to construct a nomogram with the aim of creating a quantitative method for the possibility prediction of the OS at 1, 3, and 5 years for patients with BC (Figure 6A). In the calibration curve, diagonal line (ideal model) represented the best prediction. Nomogram-predicted probability of survival was plotted on the *x*-axis meanwhile the actual survival was plotted on the *y*-axis. The higher the coincidence degree of the fitting line (red line) and the diagonal line was the better performance the nomogram exhibited. According to the calibration curve, the nomogram owned fine prediction effectiveness compared with the ideal model especially for nomogram's 1-year or 3-year OS estimates (Figure 6B-D). In the nomogram, lower total points indicated a worse outcome. The nomogram showed high accuracy as the C-index and AUC suggested. This nomogram could effectively predict OS of patients with BC by using TGCA-BLCA data (C-index: 0.671; AUC: 0.707; Figure 7A) and GSE13507 (C-index: 0.666; AUC: 0.632; Figure 7C). The C-index was 0.772 and AUC was 0.759 accurately (based on GSE13507; Figure 7B), which determined that the nomogram also performed well to predict CSS as well as PFS (E-MTAB-4321: C-index: 0.708; AUC: 0.704; Figure 7D).

### Identification of eight-gene signature associated biological pathways

**Figure 8:**
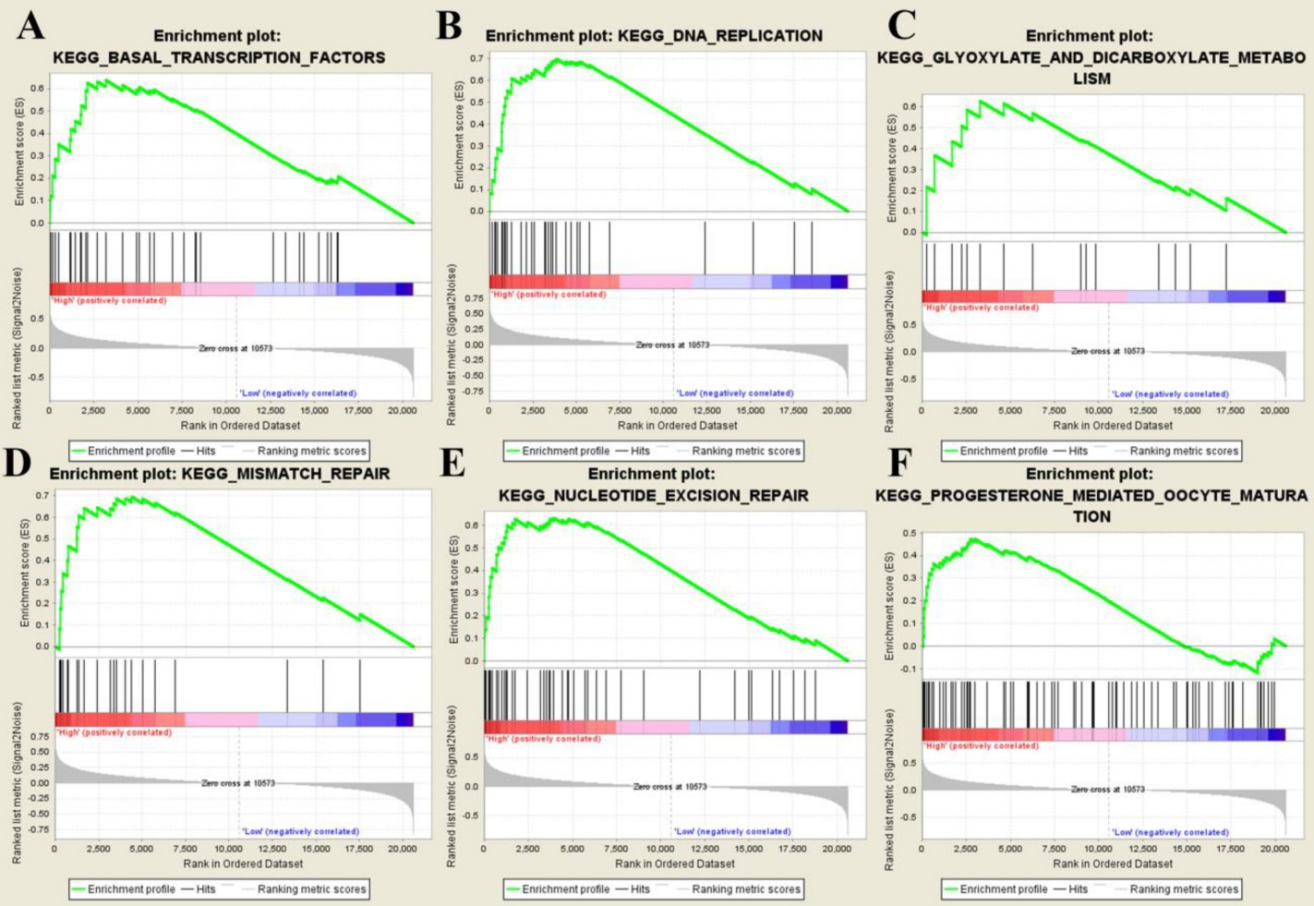
Gene set enrichment analysis. The six significant KEGG pathways including basal transcription factors (**A**), DNA replication (**B**), glyoxylate and dicarboxylate metabolism (**C**), mismatch repair (**D**), nucleotide excision repair (**E**), and progesterone-mediated oocyte maturation (**F**).

After splitting samples into high- and low- risk groups, we further performed GSEA based on GSE31684. Six risk score-related KEGG signaling pathways including basal transcription factors, DNA replication, glyoxylate and dicarboxylate metabolism, mismatch repair, nucleotide excision repair, and progesterone-mediated oocyte maturation were enriched as shown in Figure 8.

## Discussion

As the 10th most common form of cancer worldwide, BC carries a poor prognosis with an estimated 549,000 new cases and 200,000 deaths 1. Until now, cystoscopy is still the golden standard for diagnosis of BC 22. BC has caused a lot of troubles; the most major problem is that BC is of high rate of recurrence. 30-70% of BCs will relapse as reported 23. Thus, we aimed to screen several effective biomarkers and further establish a multi-gene signature for the prognosis of patients with BC.

With the development of high-throughput sequencing and bioinformatics, more and more bioinformatics methods have been used to develop novel biomarkers associated with tumor progression 24, survival 25 or prognosis 26 of malignancies, which might greatly aid the early diagnosis and evaluation of prognosis in malignant tumors. Among these methods, WGCNA has been successfully applied in the identification of novel diagnostic and prognostic biomarkers for malignancies 27, 28. We have made great efforts to apply this method in finding out prognosis biomarkers of patients with BC in our previous study 29. Therefore, we started with identifying key modules in this study by constructing a co-expression network and 12 genes associated with overall survival of BC were further selected by using Kaplan-Meier Plotter. Considering that a lot of studies have focused on establishing a multi-gene signature rather than using a single biomarker as a diagnostic or prognostic biomarker in present 30, 31, we attempted to develop a multi-gene signature in this study. LASSO is a more popular approach compared with Cox proportional hazard regression analysis for constructing prognostic gene signature models, with an advantage of preventing overfitting 32. Thus, based on the 12 biomarkers, we established an eight-gene signature which could predict prognosis of BC patients by constructed a LASSO Cox model. We further validated this signature by performing survival analysis and ROC analysis based on three other independent datasets (test set, GSE13507, E-MTAB-4321). We firstly divided patients into high- and low- risk groups according to the risk score of each patient. In the training set, the eight-gene-based classifier had the ability to distinguish the high-risk group patients from those in the low-risk group effectively. The results of survival analysis and ROC curve in the validation datasets were consistent with our findings in the training set, which made our results convincing.

Previous study had proved that tumor stage was significant associated with patient prognosis 33. The incidence of BC increased with age, with high risk age from 50 to 70 34. Our study indicated that both of tumor stage and age were significant prognostic features by performing Cox regression analysis using the TCGA-BLCA data, which was consistent with the results from previous studies. Interestingly, we found that this signature was not only independent of tumor stage and age, but also was associated with OS even had been adjusted by other clinical features. These results demonstrated that the signature could distinguish high- and low- risk groups well, which might have great predictive value of prognosis for patients with BC.

The biological role of genes in the multi-gene signature was explored by performing GSEA analysis. Six KEGG pathways were finally identified including basal transcription factors, DNA replication, glyoxylate and dicarboxylate metabolism, mismatch repair, nucleotide excision repair, and progesterone mediated oocyte maturation. We further explore genes in this signature by carrying out a literature review. Abnormal spindle microtubule assembly (ASPM), the human ortholog of the Drosophila melanogaster 'abnormal spindle' gene (asp), was the most common mutant gene associated with microcephaly primary type 5 35. ASPM was essential for normal mitotic spindle function, which could correctly guide the movement of spindles and maintain symmetric division of cytoplasm in mitosis 36. ASPM was found to up-regulate in some malignancies, such as breast cancer and gastric cancer 37. Some studies proved that the up-regulation of ASPM was associated with invasion, recurrence and poor prognosis of tumors 38, 39. Chromosome 4 open reading frame 46 (C4orf46), also known as renal cancer differentiation gene 1 (RCDG1), encoded a small, conserved protein of unknown function which was expressed in some tissues 40. RCDG1 was significantly down-regulated in renal cell carcinoma (RCC) suggested by a recent study 40. As for cyclin B1 (CCNB1), the protein encoded by cyclin B1 (CCNB1) was a regulatory protein involved in mitosis, which was necessary for proper control of the G2/M transition phase in cell cycle 41. CCNB1 could combine with p34 (cdc2) and further form the maturation-promoting factor (MPF) 42. As for diaphanous related formin 3 (DIAPH3), the protein encoded by this gene was a binding protein of actin, which participated in composing cytoskeleton proteins 43. DIAPH3 played an important role in the infiltration and metastasis of cancerous cells, which might be a potential therapeutic target for cancer treatment 44. Myeloid leukemia factor 1 (MLF1) encoded an oncoprotein which was associated with the phenotypic determination of hemopoietic cells 45. Previous study had proved that the translocations between MLF1 and nucleophosmin were related to myelodyplastic syndrome and acute myeloid 46. Mitochondrial fission regulator 2 (MTFR2) was poor studied in malignancies, a recent study identified this gene as one of the most correlated genes to dual specificity protein kinase TTK (TTK) in glioblastoma (GBM) 47. Expression of TTK was up-regulated in GBM, which was thought to be associated with poor prognosis of patients with GBM 47. NOP2/Sun RNA methyltransferase 6 (NSUN6) was a human RNA methyltransferase that catalyzed formation of m5C72 in specific tRNAs 48. NSUN6 localized to the cytoplasm, which was largely colocalized with marker proteins for the Golgi apparatus and pericentriolar matrix 49. Opa interacting protein 5 (OIP5) was a member of cancer-testis antigen (CTA) family, which might be a novel therapeutic target for cancer therapy because of the high expression of this gene in colorectal cancer 49, lung cancer 50, and esophageal cancer 50. Protein encoded by OIP5 was essential for recruitment of centromere protein A (CENPA) by the mediator Holliday junction recognition protein in centromeres 51.

Although our conclusions have been well validated by multiple independent data sets, we have not further validated our conclusions through large-scale prospective clinical and molecular biology experiments, which we believe may be the largest limitation in this study. So, we will conduct large-scale prospective clinical trials and molecular biology experiments in subsequent studies to further confirm our conclusions and related molecular biological mechanisms.

## Conclusions

To sum up, we have presented a systematic and comprehensive analysis for data from TCGA, GEO, and ArrayExpress databases by using WGCNA and LASSO as the main methods. We identified 12 genes associated with overall survival of BC patients and further developed an eight-gene signature which was of great value to predict prognosis of BC. This eight-gene-based classifier might be of great value for making prognostic evaluations. However, the eight-gene signature must be validated by using clinical trials and more advanced methods in bioinformatics field.

## Supplementary Material

Supplementary figures and tables.

## Figures and Tables

**Table 1:** Clinical characteristics of patients with BC in each study.

Characteristics	Training dataset	Internal test dataset	Entire TCGA dataset	GSE31684	GSE13507	E-MTAB-4321
**Number of patients**	195	196	391	93	165	476
Age(median, IQR*)	68(61, 76)	70(61, 77)	69(61, 77)	69(62, 77)	65(59,74)	69(62, 76)
≥ 65	120	136	256	67	96	323
< 65	74	60	134	26	69	153
NA	1	0	1	0		0
**Gender**						
Male	142	145	287	68	135	367
Female	53	51	104	25	30	109
**Stage**						
I	1	1	2	15	104	457
II	55	62	117	17	31	16
III	70	69	139	42	19	0
IV	67	64	131	19	11	0
NA	2	0	2	0	0	3
**Grade**						
Low	5	7	12	6	105	277
High	187	189	376	87	60	192
NA	3	0	3	0	0	7

*IQR: interquartile range.

## Abbreviations

ASPM: Abnormal spindle microtubule assembly
AUC: Area under curve
BC: Bladder cancer
BP: Biological process
C4orf46: Chromosome 4 open reading frame 46
CCNB1: cyclin B1
CENPA: Centromere protein A
CSS: Cancer-specific survival
CTA: cancer-testis antigen
DCA: Decision curve analysis
DIAPH3: Diaphanous related formin 3
GBM: glioblastoma
GEO: Gene Expression Omnibus
GO: Gene Ontology
GS: Gene Significance
GSEA: Gene set enrichment analysis
KEGG: Kyoto Encyclopedia of Genes and Genomes
LASSO: Least absolute shrinkage and selection operator
MLF1: Myeloid leukemia factor 1
MPF: maturation-promoting factor
MS: Module Significance
MTFR2: Mitochondrial fission regulator 2
NSUN6: NOP2/Sun RNA methyltransferase 6
OIP5: Opa interacting protein 5
OS: Overall survival
Pt: Threshold Probability
RCDG1: Renal cancer differentiation gene 1
ROC: Receiver operating characteristic
RS: Risk score
TCGA: The Cancer Genome Atlas
WGCNA: Weighted gene co-expression network analysis
