# Supplementary material for: Construction and validation of an eight-gene signature with great prognostic value in bladder cancer
Source: J Cancer. 2020 Jan 17;11(7):1768–79. doi: 10.7150/jca.38741 (PMC7052873; doi:10.7150/jca.38741)
Supplement: Supplementary file 1 — Supplementary figures and tables. [file jcav11p1768s1.pdf]

Supplementary materials:

Supplementary figures:

Figure S1: The clustering was based on the expression data of GSE31684. The top 5,000 genes with the highest SD values were used for the analysis by WGCNA. The color intensity was proportional to gender, survival months (survival time), stage, grade and age.

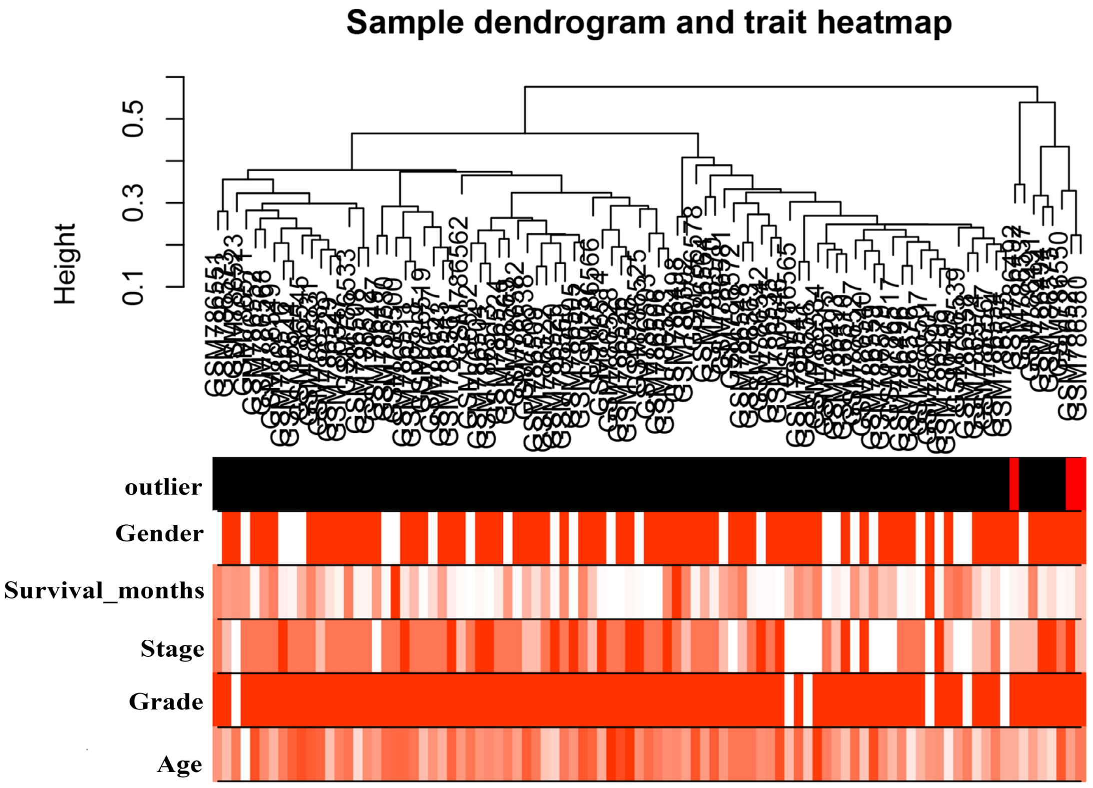

Figure S2: Determination of soft-thresholding power in the weighted gene co-expression network analysis (WGCNA). (A) Analysis of the scale-free fit index for various soft-thresholding powers (β). (B) Analysis of the mean connectivity for various soft-thresholding powers. (C) Histogram of connectivity distribution when $\beta = 4$. (D) Checking the scale free topology when $\beta = 4$.

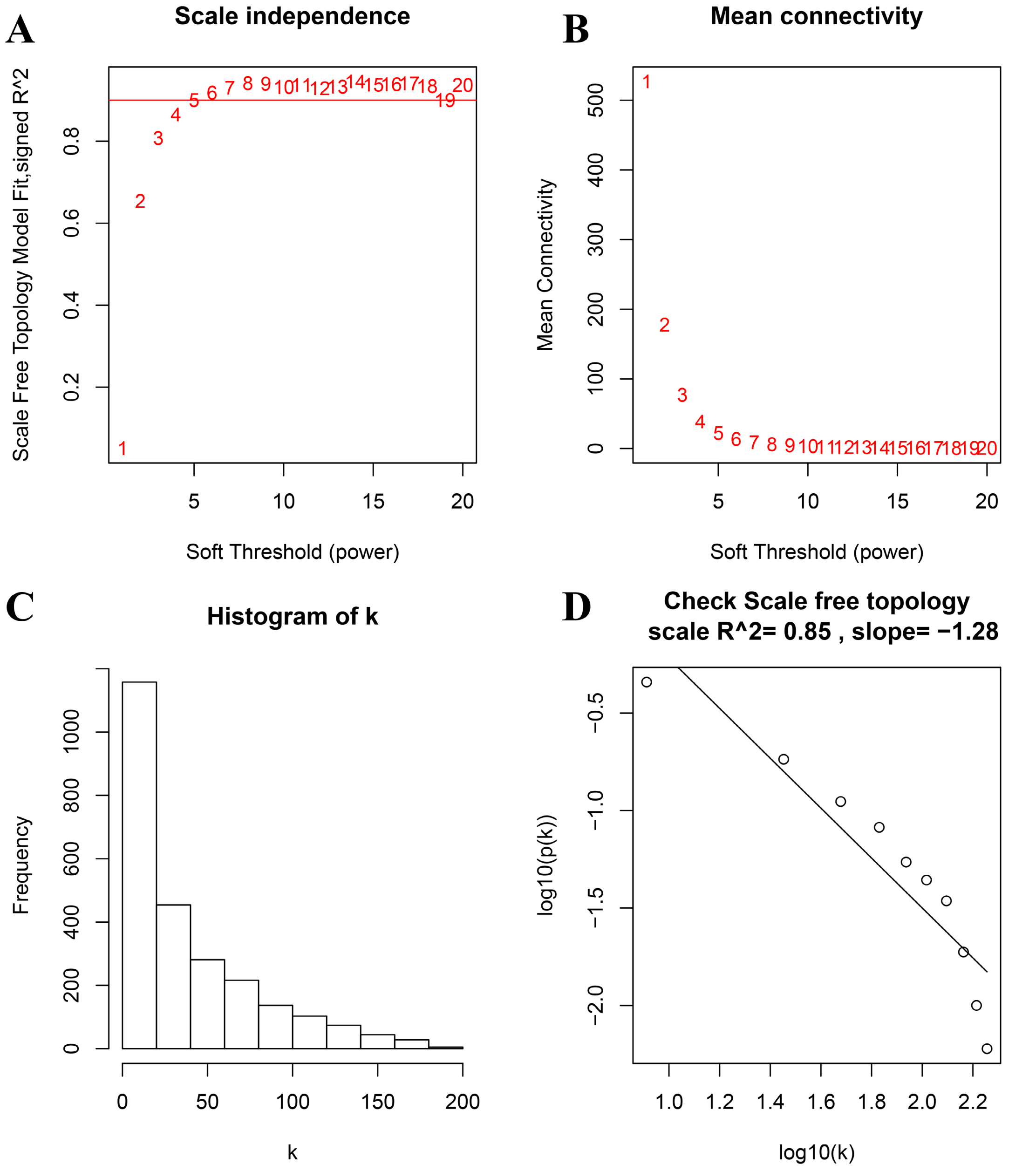

Figure S3: The cluster dendrogram of genes in GSE31684. Each branch in the figure represents one gene, and every color below represents one co-expression module.

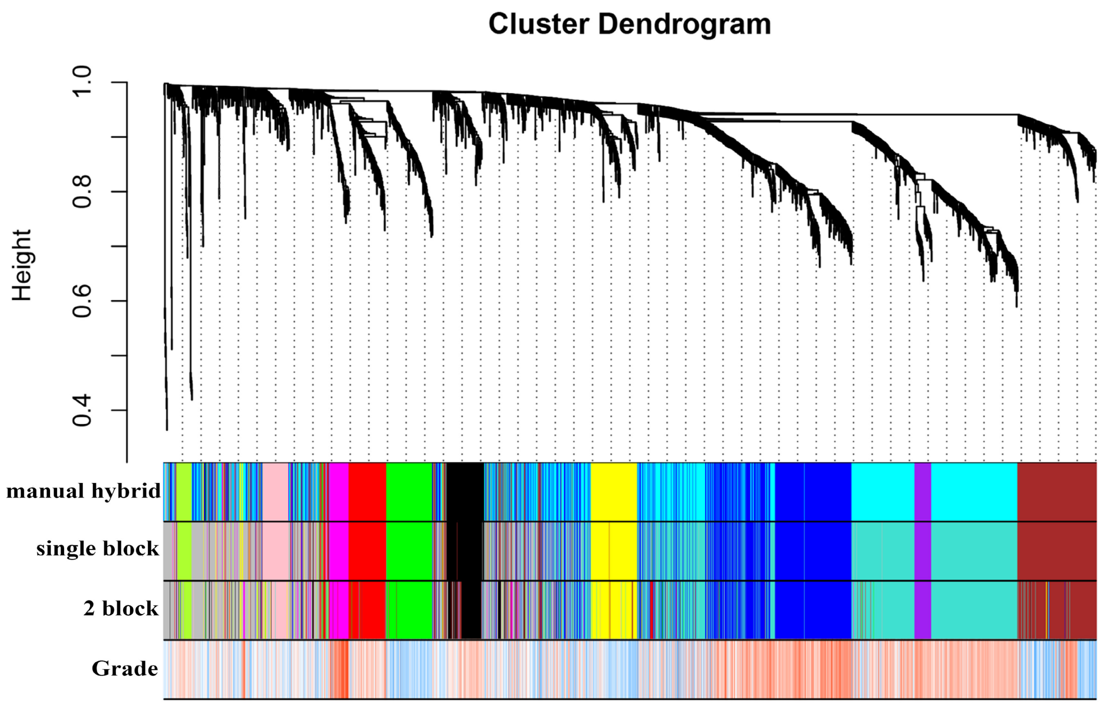

Figure S4: (A) Interaction relationship analysis of co-expression genes. Different colors of horizontal axis and vertical axis represent different modules. The brightness of yellow in the middle represents the degree of connectivity of different modules. There was no significant difference in interactions among different modules, indicating a high-scale independence degree among these modules. (B) Classical MDS plot whose input is the TOM dissimilarity. Each dot (gene) is colored by the module assignment.

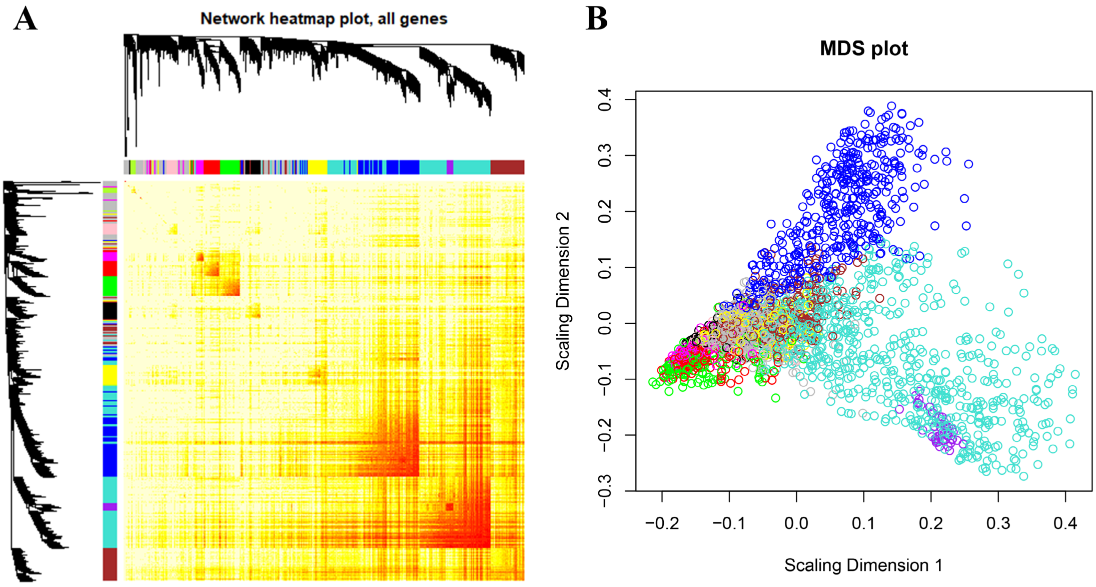

Supplementary tables:

Table S1: Gene Ontology (GO) biological processes of genes in key module.

ID	Description	GeneRatio	BgRatio	pvalue	p.adjust	qvalue	Count
GO:0048285	organelle fission	18/65	457/17653	3.18E-14	1.34E-11	9.90E-12	18
GO:0007059	chromosome segregation	17/65	358/17653	8.77E-15	8.92E-12	6.61E-12	17
GO:0000280	nuclear division	16/65	411/17653	1.27E-12	1.85E-10	1.37E-10	16
GO:0140014	mitotic nuclear division	15/65	269/17653	3.94E-14	1.34E-11	9.90E-12	15
GO:0000819	sister chromatid segregation	14/65	239/17653	1.57E-13	3.19E-11	2.37E-11	14
GO:0098813	nuclear chromosome segregation	14/65	307/17653	4.74E-12	5.35E-10	3.97E-10	14
GO:1902850	microtubule cytoskeleton organization involved in mitosis	12/65	132/17653	5.28E-14	1.34E-11	9.96E-12	12
GO:0007051	spindle organization	12/65	183/17653	2.68E-12	3.40E-10	2.52E-10	12
GO:0007052	mitotic spindle organization	11/65	113/17653	3.01E-13	5.11E-11	3.79E-11	11
GO:1901987	regulation of cell cycle phase transition	11/65	454/17653	7.39E-07	4.77E-05	3.54E-05	11
GO:0000070	mitotic sister chromatid segregation	10/65	152/17653	1.98E-10	2.02E-08	1.50E-08	10
GO:0051783	regulation of nuclear division	10/65	184/17653	1.29E-09	1.19E-07	8.85E-08	10
GO:0045787	positive regulation of cell cycle	10/65	377/17653	1.10E-06	5.61E-05	4.15E-05	10
GO:1901990	regulation of mitotic cell cycle phase transition	10/65	419/17653	2.85E-06	0.000107	7.94E-05	10
GO:0007088	regulation of mitotic nuclear division	9/65	161/17653	7.21E-09	6.11E-07	4.53E-07	9
GO:0090068	positive regulation of cell cycle process	9/65	277/17653	7.51E-07	4.77E-05	3.54E-05	9
GO:0007062	sister chromatid cohesion	8/65	134/17653	3.13E-08	2.45E-06	1.82E-06	8
GO:0007050	cell cycle arrest	8/65	250/17653	3.64E-06	0.000124	9.19E-05	8
GO:0044843	cell cycle G1/S phase transition	8/65	295/17653	1.22E-05	0.000303	0.000225	8
GO:0051225	spindle assembly	7/65	122/17653	3.16E-07	2.30E-05	1.70E-05	7
GO:0000910	cytokinesis	7/65	172/17653	3.19E-06	0.000116	8.59E-05	7
GO:0000086	G2/M transition of mitotic cell cycle	7/65	250/17653	3.63E-05	0.00077	0.00057	7
GO:0044839	cell cycle G2/M phase transition	7/65	267/17653	5.51E-05	0.001099	0.000815	7
GO:0000082	G1/S transition of mitotic cell cycle	7/65	276/17653	6.79E-05	0.001303	0.000966	7
GO:0033044	regulation of chromosome organization	7/65	311/17653	0.000143	0.002202	0.001632	7
GO:0051656	establishment of organelle localization	7/65	450/17653	0.001299	0.010401	0.007708	7
GO:0051983	regulation of chromosome segregation	6/65	100/17653	1.79E-06	7.59E-05	5.62E-05	6
GO:0071156	regulation of cell cycle arrest	6/65	113/17653	3.66E-06	0.000124	9.19E-05	6
GO:0007093	mitotic cell cycle checkpoint	6/65	166/17653	3.30E-05	0.000714	0.000529	6
GO:0000075	cell cycle checkpoint	6/65	225/17653	0.000177	0.002686	0.00199	6
GO:1901991	negative regulation of mitotic cell cycle phase transition	6/65	236/17653	0.000229	0.003025	0.002242	6

GO:0051321	meiotic cell cycle	6/65	246/17653	0.000286	0.003513	0.002603	6
GO:1901988	negative regulation of cell cycle phase transition	6/65	256/17653	0.000354	0.004188	0.003104	6
GO:0072331	signal transduction by p53 class mediator	6/65	272/17653	0.000488	0.0054	0.004002	6
GO:0045930	negative regulation of mitotic cell cycle	6/65	319/17653	0.001121	0.009497	0.007038	6
GO:0010948	negative regulation of cell cycle process	6/65	350/17653	0.001797	0.013339	0.009885	6
GO:0030071	regulation of mitotic metaphase/anaphase transition	5/65	48/17653	8.77E-07	5.21E-05	3.86E-05	5
GO:1902099	regulation of metaphase/anaphase transition of cell cycle	5/65	49/17653	9.74E-07	5.21E-05	3.86E-05	5
GO:0007091	metaphase/anaphase transition of mitotic cell cycle	5/65	51/17653	1.19E-06	5.78E-05	4.28E-05	5
GO:0044784	metaphase/anaphase transition of cell cycle	5/65	52/17653	1.32E-06	6.08E-05	4.51E-05	5
GO:0010965	regulation of mitotic sister chromatid separation	5/65	54/17653	1.59E-06	7.04E-05	5.22E-05	5
GO:0051306	mitotic sister chromatid separation	5/65	56/17653	1.91E-06	7.78E-05	5.77E-05	5
GO:1905818	regulation of chromosome separation	5/65	58/17653	2.28E-06	8.92E-05	6.61E-05	5
GO:0033047	regulation of mitotic sister chromatid segregation	5/65	65/17653	4.03E-06	0.000132	9.80E-05	5
GO:0000281	mitotic cytokinesis	5/65	67/17653	4.69E-06	0.000144	0.000107	5
GO:0090307	mitotic spindle assembly	5/65	67/17653	4.69E-06	0.000144	0.000107	5
GO:0033045	regulation of sister chromatid segregation	5/65	76/17653	8.74E-06	0.000261	0.000194	5
GO:0051303	establishment of chromosome localization	5/65	77/17653	9.32E-06	0.000271	0.000201	5
GO:0050000	chromosome localization	5/65	78/17653	9.93E-06	0.000277	0.000206	5
GO:0031145	anaphase-promoting complex-dependent catabolic process	5/65	80/17653	1.12E-05	0.000293	0.000217	5
GO:0051304	chromosome separation	5/65	80/17653	1.12E-05	0.000293	0.000217	5
GO:0061640	cytoskeleton-dependent cytokinesis	5/65	81/17653	1.20E-05	0.000303	0.000225	5
GO:0000079	regulation of cyclin-dependent protein serine/threonine kinase activity	5/65	88/17653	1.79E-05	0.000424	0.000314	5
GO:0071158	positive regulation of cell cycle arrest	5/65	88/17653	1.79E-05	0.000424	0.000314	5
GO:1904029	regulation of cyclin-dependent protein kinase activity	5/65	93/17653	2.34E-05	0.00053	0.000392	5
GO:0030330	DNA damage response, signal	5/65	110/17653	5.26E-05	0.00107	0.000793	5

	transduction by p53 class mediator						
GO:0042770	signal transduction in response to DNA damage	5/65	134/17653	0.000134	0.002148	0.001592	5
GO:0045931	positive regulation of mitotic cell cycle	5/65	148/17653	0.000213	0.00291	0.002157	5
GO:0140013	meiotic nuclear division	5/65	174/17653	0.00045	0.005141	0.00381	5
GO:1903046	meiotic cell cycle process	5/65	189/17653	0.000655	0.006532	0.004841	5
GO:0006323	DNA packaging	5/65	208/17653	0.001007	0.008893	0.006591	5
GO:1902749	regulation of cell cycle G2/M phase transition	5/65	216/17653	0.001192	0.009853	0.007302	5
GO:0051383	kinetochore organization	4/65	21/17653	9.56E-07	5.21E-05	3.86E-05	4
GO:0016572	histone phosphorylation	4/65	37/17653	1.01E-05	0.000277	0.000206	4
GO:0007080	mitotic metaphase plate congression	4/65	44/17653	2.03E-05	0.00047	0.000348	4
GO:0034508	centromere complex assembly	4/65	53/17653	4.28E-05	0.000888	0.000658	4
GO:0051310	metaphase plate congression	4/65	57/17653	5.71E-05	0.001117	0.000828	4
GO:0006977	DNA damage response, signal transduction by p53 class mediator resulting in cell cycle arrest	4/65	62/17653	7.95E-05	0.001498	0.00111	4
GO:0072431	signal transduction involved in mitotic G1 DNA damage checkpoint	4/65	63/17653	8.47E-05	0.001538	0.00114	4
GO:1902400	intracellular signal transduction involved in G1 DNA damage checkpoint	4/65	63/17653	8.47E-05	0.001538	0.00114	4
GO:0072413	signal transduction involved in mitotic cell cycle checkpoint	4/65	65/17653	9.57E-05	0.001623	0.001203	4
GO:1902402	signal transduction involved in mitotic DNA damage checkpoint	4/65	65/17653	9.57E-05	0.001623	0.001203	4
GO:1902403	signal transduction involved in mitotic DNA integrity checkpoint	4/65	65/17653	9.57E-05	0.001623	0.001203	4
GO:0031571	mitotic G1 DNA damage checkpoint	4/65	71/17653	0.000135	0.002148	0.001592	4
GO:0044819	mitotic G1/S transition checkpoint	4/65	71/17653	0.000135	0.002148	0.001592	4
GO:0044783	G1 DNA damage checkpoint	4/65	72/17653	0.000143	0.002202	0.001632	4
GO:0072401	signal transduction involved in DNA integrity checkpoint	4/65	78/17653	0.000195	0.00281	0.002083	4
GO:0072422	signal transduction involved in DNA damage checkpoint	4/65	78/17653	0.000195	0.00281	0.002083	4
GO:0072395	signal transduction involved in cell cycle checkpoint	4/65	79/17653	0.000204	0.002848	0.002111	4
GO:0048477	oogenesis	4/65	80/17653	0.000215	0.00291	0.002157	4
GO:0006890	retrograde vesicle-mediated transport, Golgi to ER	4/65	83/17653	0.000247	0.003144	0.00233	4
GO:1901989	positive regulation of cell cycle	4/65	87/17653	0.000296	0.003587	0.002658	4

	phase transition						
GO:0019886	antigen processing and presentation of exogenous peptide antigen via MHC class II	4/65	98/17653	0.000466	0.005213	0.003863	4
GO:0002495	antigen processing and presentation of peptide antigen via MHC class II	4/65	101/17653	0.000523	0.005607	0.004155	4
GO:0002504	antigen processing and presentation of peptide or polysaccharide antigen via MHC class II	4/65	102/17653	0.000543	0.005691	0.004217	4
GO:0044773	mitotic DNA damage checkpoint	4/65	107/17653	0.00065	0.006532	0.004841	4
GO:0044774	mitotic DNA integrity checkpoint	4/65	113/17653	0.000798	0.007728	0.005727	4
GO:2000134	negative regulation of G1/S transition of mitotic cell cycle	4/65	116/17653	0.00088	0.008063	0.005975	4
GO:1902807	negative regulation of cell cycle G1/S phase transition	4/65	123/17653	0.001095	0.009354	0.006932	4
GO:2001251	negative regulation of chromosome organization	4/65	123/17653	0.001095	0.009354	0.006932	4
GO:0007292	female gamete generation	4/65	127/17653	0.001232	0.010026	0.00743	4
GO:2000241	regulation of reproductive process	4/65	140/17653	0.001764	0.013191	0.009776	4
GO:0051382	kinetochore assembly	3/65	16/17653	2.58E-05	0.00057	0.000422	3
GO:0001556	oocyte maturation	3/65	24/17653	9.13E-05	0.001623	0.001203	3
GO:1902751	positive regulation of cell cycle G2/M phase transition	3/65	26/17653	0.000117	0.001944	0.001441	3
GO:0007094	mitotic spindle assembly checkpoint	3/65	31/17653	0.000199	0.00281	0.002083	3
GO:0071173	spindle assembly checkpoint	3/65	31/17653	0.000199	0.00281	0.002083	3
GO:0071174	mitotic spindle checkpoint	3/65	31/17653	0.000199	0.00281	0.002083	3
GO:0045841	negative regulation of mitotic metaphase/anaphase transition	3/65	32/17653	0.000219	0.00293	0.002171	3
GO:0008608	attachment of spindle microtubules to kinetochore	3/65	33/17653	0.00024	0.003093	0.002292	3
GO:1902100	negative regulation of metaphase/anaphase transition of cell cycle	3/65	33/17653	0.00024	0.003093	0.002292	3
GO:0031577	spindle checkpoint	3/65	34/17653	0.000263	0.003299	0.002445	3
GO:2000816	negative regulation of mitotic sister chromatid separation	3/65	35/17653	0.000287	0.003513	0.002603	3
GO:1905819	negative regulation of chromosome separation	3/65	36/17653	0.000312	0.003732	0.002766	3
GO:0033048	negative regulation of mitotic sister chromatid segregation	3/65	38/17653	0.000367	0.004285	0.003176	3
GO:0033046	negative regulation of sister chromatid segregation	3/65	40/17653	0.000427	0.004936	0.003658	3
GO:0051985	negative regulation of chromosome	3/65	41/17653	0.00046	0.005193	0.003849	3

	segregation						
GO:0034080	CENP-A containing nucleosome assembly	3/65	43/17653	0.000529	0.005607	0.004155	3
GO:0051445	regulation of meiotic cell cycle	3/65	43/17653	0.000529	0.005607	0.004155	3
GO:0061641	CENP-A containing chromatin organization	3/65	43/17653	0.000529	0.005607	0.004155	3
GO:0048599	oocyte development	3/65	44/17653	0.000567	0.005879	0.004357	3
GO:0031055	chromatin remodeling at centromere	3/65	45/17653	0.000605	0.006157	0.004563	3
GO:0045839	negative regulation of mitotic nuclear division	3/65	48/17653	0.000732	0.007229	0.005357	3
GO:0009994	oocyte differentiation	3/65	49/17653	0.000778	0.007606	0.005637	3
GO:0006336	DNA replication-independent nucleosome assembly	3/65	53/17653	0.000979	0.008887	0.006586	3
GO:0034724	DNA replication-independent nucleosome organization	3/65	54/17653	0.001034	0.008984	0.006658	3
GO:0043486	histone exchange	3/65	57/17653	0.00121	0.009922	0.007353	3
GO:0051784	negative regulation of nuclear division	3/65	58/17653	0.001272	0.01027	0.007611	3
GO:1901607	alpha-amino acid biosynthetic process	3/65	63/17653	0.001616	0.012547	0.009298	3
GO:0051256	mitotic spindle midzone assembly	2/65	10/17653	0.000589	0.006055	0.004487	2
GO:0000022	mitotic spindle elongation	2/65	12/17653	0.00086	0.007955	0.005895	2
GO:0006563	L-serine metabolic process	2/65	12/17653	0.00086	0.007955	0.005895	2
GO:0035404	histone-serine phosphorylation	2/65	12/17653	0.00086	0.007955	0.005895	2
GO:0051255	spindle midzone assembly	2/65	12/17653	0.00086	0.007955	0.005895	2
GO:0055015	ventricular cardiac muscle cell development	2/65	12/17653	0.00086	0.007955	0.005895	2
GO:0000212	meiotic spindle organization	2/65	13/17653	0.001014	0.008893	0.006591	2
GO:0051231	spindle elongation	2/65	13/17653	0.001014	0.008893	0.006591	2
GO:0051315	attachment of mitotic spindle microtubules to kinetochore	2/65	13/17653	0.001014	0.008893	0.006591	2
GO:0090266	regulation of mitotic cell cycle spindle assembly checkpoint	2/65	14/17653	0.001181	0.009842	0.007294	2
GO:1903504	regulation of mitotic spindle checkpoint	2/65	14/17653	0.001181	0.009842	0.007294	2
GO:0090231	regulation of spindle checkpoint	2/65	15/17653	0.001359	0.010798	0.008002	2
GO:0002082	regulation of oxidative phosphorylation	2/65	16/17653	0.00155	0.012122	0.008984	2
GO:0007100	mitotic centrosome separation	2/65	16/17653	0.00155	0.012122	0.008984	2
GO:0009070	serine family amino acid biosynthetic process	2/65	17/17653	0.001752	0.013191	0.009776	2
GO:0051299	centrosome separation	2/65	17/17653	0.001752	0.013191	0.009776	2
GO:0051782	negative regulation of cell division	2/65	17/17653	0.001752	0.013191	0.009776	2

GO:0055012	ventricular cardiac muscle cell differentiation	2/65	17/17653	0.001752	0.013191	0.009776	2

Table S2: Survival analysis of hub genes in co-expression network.

**Gene symbol**	**logrank *P***	**HR**
ANLN	0.0036	1.54 (1.15-2.07)
ASPM	0.033	1.39 (1.03-1.88)
ATAD2	0.085	0.75 (0.53-1.04)
AURKA	0.074	1.34 (0.97-1.85)
BIRC5	0.13	1.28 (0.93-1.76)
C4orf46	0.018	0.7 (0.52-0.94)
CCNA2	0.18	1.24 (0.91-1.68)
CCNB1	0.0044	1.54 (1.14-2.08)
CDC20	0.011	1.52 (1.1-2.1)
CDC25A	0.22	1.2 (0.89-1.62)
CDK1	0.11	0.78 (0.58-1.06)
CDK5R1	0.088	1.32 (0.96-1.83)
CDKN3	0.061	1.33 (0.99-1.79)
CENPA	0.32	1.19 (0.85-1.66)
CENPE	0.26	1.19 (0.88-1.6)
CENPN	0.14	1.32 (0.91-1.9)
CEP55	0.2	0.82 (0.61-1.11)
CHAC2	0.22	0.82 (0.6-1.13)
DEPDC1	0.14	1.27 (0.92-1.75)
DEPDC1B	0.071	0.76 (0.56-1.03)
DIAPH3	0.0062	1.55 (1.13-2.12)
DLGAP5	0.021	1.41 (1.05-1.89)
FAM64A	0.089	0.76 (0.55-1.04)
FAM72A	0.054	1.36 (0.99-1.85)
FOXM1	0.22	1.2 (0.89-1.62)
GGH	0.07	1.31 (0.98-1.76)
HMMR	0.016	1.48 (1.07-2.05)
KIF11	0.28	1.19 (0.87-1.63)
KIF23	0.13	0.8 (0.59-1.07)
KIF4A	0.18	0.82 (0.61-1.1)
MCM10	0.11	1.29 (0.94-1.77)
MKI67	0.45	1.12 (0.83-1.5)
MLF1	0.045	1.36 (1.01-1.83)
MTFR2	0.04	0.73 (0.54-0.99)
NETO2	0.13	1.27 (0.93-1.74)
NSUN6	0.0026	0.6 (0.43-0.84)
NUF2	0.11	0.77 (0.56-1.06)
ODC1	0.14	1.27 (0.92-1.74)
OIP5	0.033	1.39 (1.03-1.87)

PBK	0.08	1.3 (0.97-1.75)
PSRC1	0.22	1.21 (0.89-1.63)
SHCBP1	0.3	1.21 (0.85-1.73)
SPC25	0.22	0.83 (0.62-1.12)
TMEM38B	0.12	1.29 (0.94-1.76)
TRIP13	0.37	1.16 (0.83-1.63)
